# Modeling the Dynamics and Migratory Pathways of Virus-Specific Antibody-Secreting Cell Populations in Primary Influenza Infection

**DOI:** 10.1371/journal.pone.0104781

**Published:** 2014-08-29

**Authors:** Hongyu Miao, Mark Y. Sangster, Alexandra M. Livingstone, Shannon P. Hilchey, Le Zhang, David J. Topham, Tim R. Mosmann, Jeanne Holden-Wiltse, Alan S. Perelson, Hulin Wu, Martin S. Zand

**Affiliations:** 1 Department of Biostatistics and Computational Biology, University of Rochester Medical Center, Rochester, New York, United States of America; 2 David H. Smith Center for Vaccine Biology & Immunology, Department of Microbiology and Immunology, University of Rochester Medical Center, Rochester, New York, United States of America; 3 Department of Medicine, University of Rochester Medical Center, Rochester, New York, United States of America; 4 Theoretical Biology and Biophysics, Los Alamos National Laboratory, Los Alamos, New Mexico, United States of America; University of Melbourne, Australia

## Abstract

The B cell response to influenza infection of the respiratory tract contributes to viral clearance and establishes profound resistance to reinfection by related viruses. Numerous studies have measured virus-specific antibody-secreting cell (ASC) frequencies in different anatomical compartments after influenza infection and provided a general picture of the kinetics of ASC formation and dispersion. However, the dynamics of ASC populations are difficult to determine experimentally and have received little attention. Here, we applied mathematical modeling to investigate the dynamics of ASC growth, death, and migration over the 2-week period following primary influenza infection in mice. Experimental data for model fitting came from high frequency measurements of virus-specific IgM, IgG, and IgA ASCs in the mediastinal lymph node (MLN), spleen, and lung. Model construction was based on a set of assumptions about ASC gain and loss from the sampled sites, and also on the directionality of ASC trafficking pathways. Most notably, modeling results suggest that differences in ASC fate and trafficking patterns reflect the site of formation and the expressed antibody class. Essentially all early IgA ASCs in the MLN migrated to spleen or lung, whereas cell death was likely the major reason for IgM and IgG ASC loss from the MLN. In contrast, the spleen contributed most of the IgM and IgG ASCs that migrated to the lung, but essentially none of the IgA ASCs. This finding points to a critical role for regional lymph nodes such as the MLN in the rapid generation of IgA ASCs that seed the lung. Results for the MLN also suggest that ASC death is a significant early feature of the B cell response. Overall, our analysis is consistent with accepted concepts in many regards, but it also indicates novel features of the B cell response to influenza that warrant further investigation.

## Introduction

The antibody (Ab) response against influenza infection involves activation and progressive differentiation of virus-specific B cells into Ab-secreting cells (ASCs). A similar process occurs during intramuscular influenza vaccination. In both cases, Ab-mediated immunity develops after influenza-specific B cells produce high affinity Abs, most importantly against the haemagglutinin (HA) protein responsible for viral binding to target respiratory epithelial cells. B cells activated by influenza infection or vaccination may develop into ASCs secreting the IgM Ab class, or may undergo class switching during the differentiation process and form IgG or IgA ASCs. The Ab class reflects functional capabilities of the immunoglobulin molecule, such as complement activation, Fc receptor binding, and transcytosis of epithelial cells at mucosal surfaces.

Studies by several groups have characterized ASC formation during primary influenza A virus infection using murine models [1]–[5]. Influenza-specific ASCs first develop in lymph nodes that drain the respiratory tract and a day or so later in the spleen. In sites of ASC formation, a peak of IgM ASCs typically precedes increasing numbers of IgG and IgA ASCs. Influenza-specific ASC numbers in the regional lymph nodes and spleen gradually wane after clearance of infectious virus, but in the course of the response ASCs traffic to the respiratory tract and bone marrow and establish long-lasting populations. A rapid increase in serum levels of influenza-specific IgM and IgG beginning approximately 7 days after infection closely follows initial ASC formation. Serum IgM levels peak at 8–10 days and then gradually decline, reflecting the IgM ASC numbers in lymphoid tissues. However, high serum levels of IgG are maintained long-term, primarily by ASCs in the bone marrow [6], [7]. Although much has been learned, B cell dynamics in the context of primary influenza infection have not been well characterized in a quantitative manner. Specifically, we know little about the dynamics of ASC division, death and migration, the routes taken by ASCs after they migrate from sites of formation, the rates of ASC trafficking from site-to-site, and the number and source of ASCs that migrate to the site of infection in the lung.

The key dynamic parameters mentioned above are very difficult to measure experimentally. For example, direct measurement of the rate at which activated B cells transit from regional lymph nodes to bone marrow requires real-time measurement and direct tracking of labeled cells over a period of 12–24 hours in a live mouse. However, such kinetic parameters can be estimated using quantitative mathematical models. This approach has been used by other groups to estimate the survival time of free virus and virus-infected cells at particular stages of infection, the relative contributions of different Ab classes to viral clearance, and the relative importance of host lymphoid tissues in generating antiviral effector T cells that migrate to sites of infection [6], [8]–[13]. In the current study, we applied mathematical modeling to investigate the dynamics of virus-specific ASCs over the 2-week period immediately following primary influenza infection in mice. High frequency time-course measurements of IgM, IgG, and IgA ASC frequencies in a regional lymph node, the spleen, and the lung provided the basis for the development of mathematical models to describe ASC appearance, disappearance, and migration. To refine parameter estimates, semi-mechanistic models were also considered and best models were selected based on model comparison criteria. Our combination of experimental data and mathematical modeling provides a precise depiction of early ASC dynamics in the context of influenza infection.

## Materials and Methods

### Mouse Infection and Sampling

All animal experiments were performed under a protocol approved and monitored by the University Committee on Animal Research (UCAR) at the University of Rochester Medical Center, and in compliance with the United States Public Health Service (PHS) Policy on Humane Care and Use of Laboratory Animals.

C57BL/6NCr mice at 8 to 10 weeks of age were anesthetized using 2,2,2-tribromoethanol (Avertin) and then infected intranasally with 10^5^ EID_50_ H3N2 A/Hong Kong/X31 (X31) influenza virus [13]. On the day of organ harvest, mice (*n* = 9/day) were euthanized. The mediastinal lymph node (MLN) and spleen were collected and disrupted to generate single-cell suspensions. Lungs were disrupted and strained through a nylon mesh. Red blood cells were lysed using buffered ammonium chloride solution (Gey's solution). Pelleted lung cells were resuspended in 5 ml of complete minimum essential medium (cMEM), layered over 5 ml of Histopaque 1083 (Sigma, St. Louis, MO), and centrifuged for 18 min at 1,800×*g*. After centrifugation, cells at the interface were removed, washed, and resuspended in cMEM.

### ELISpot Assay

Influenza-specific ASCs were enumerated by ELISpot assay. Plates were coated with purified X31 and single cell suspensions were plated and incubated as previously described [14]. Plates were washed and alkaline phosphatase-conjugated goat anti-mouse Abs specific for IgM, IgG, or IgA (KPL, Gaithersburg, MD) were added to the appropriate wells. After overnight incubation at 4°C, plates were washed thoroughly and incubated with alkaline phosphatase substrate kit III (Vector Laboratories, Burlingame, CA) for 30 min at room temperature. Plates were washed and dried after optimal spot development and spots were counted using a CTL ImmunoSpot plate reader (Cellular Technology Limited, Cleveland, OH).

### Mathematical Models

**Figure 1:**
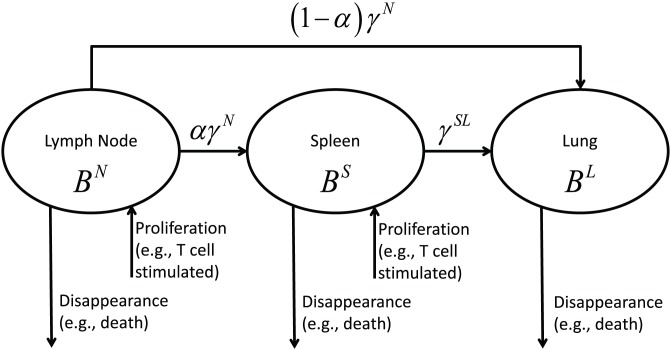
Schematic diagram of ASC migration pathways involving MLN, spleen, and lung following influenza infection.

We first developed a mechanistic ordinary differential equation (ODE) model to describe the dynamics of virus-specific ASC populations in the blood, MLN, spleen, and lung compartments following primary influenza infection. Model construction was based on a set of assumptions derived from our current understanding of the processes of ASC formation and dispersion (reviewed in [15]). To briefly outline these processes, as influenza virus replicates in the respiratory tract, B cell responses are initiated by the delivery of viral antigens to draining lymph nodes and to the spleen. Virus-specific ASC formation is largely a T cell-dependent process that involves the proliferation and differentiation of activated B cells and Ab class switching. Activated B cells can progress along an extrafollicular differentiation pathway to form short-lived ASCs, or can enter germinal center reactions where affinity maturation of expressed Abs takes place and long-lived ASCs are produced. ASCs induced by influenza infection exit the draining lymph nodes (initially via efferent lymphatics) and the spleen (directly into the blood) and are transported in the blood to establish long-lasting populations in the respiratory tract and bone marrow. Some trafficking of ASCs from responding lymph nodes to spleen is predicted because of the phase of blood-borne migration. Specific assumptions for model construction (illustrated in Fig. 1) are as follows: (i) ASC numbers in the MLN are affected by differentiation and proliferation within the lymph node, migration to spleen, migration to lung, migration to other compartments that were not experimentally measured (e.g., bone marrow), and cell death (a term used to encompass all mechanisms of cell death as well as processes resulting in loss of Ab secretion by living cells); (ii) ASC numbers in the spleen reflect differentiation and proliferation, influx from MLN, migration to lung, migration to other compartments, and loss through cell death; and (iii) ASC numbers in the lung reflect influx from both MLN and spleen, with loss due to cell death. ASC transition from a cell secreting a particular Ab class into a cell secreting a different Ab class (for example, transition from an IgM ASC to an IgG ASC) was not incorporated into our model. This is an unlikely event, since transcriptional programming for ASC formation is associated with down-regulation of Ab class switching [16]. We denote ASC numbers by *B* (for B cell-derived); superscripted letters denote the anatomical compartment (*N* for MLN, *S* for spleen, and *L* for lung) and subscripted letters denote the secreted Ab class (*M* for IgM, *G* for IgG, and *A* for IgA). For example, 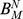 denotes the number of IgM ASCs in the MLN.

The virus-specific B cell response to influenza infection in the MLN and spleen is largely dependent on cognate help delivered by CD4 T cells. For simplicity, we do not explicitly represent the effect of T cell help, but instead parameterize the model with a general term representing the strength of B cell activation. This formulation allows the magnitude and timing of the activation strength to take any reasonable value and is thus flexible enough to allow for both cognate and non-cognate B cell help (see the stimulation strength coefficient and the time delay parameters in Model 1 below).

ASC loss from a compartment results from emigration and cell death. The rate of ASC migration via pathways involving the MLN, the spleen, or the lung is denoted by the symbol *γ*, together with a one- or two-letter superscript to identify aspects of cell migration (see Table 1). We use the symbol *δ* to denote the rate of ASC disappearance, with disappearance specifically defined as ASC loss resulting from migration to sites not explicitly included in our model (e.g., bone marrow and liver) or from cell death. The mechanistic model has exactly the same structure for IgM, IgG, and IgA ASCs, so we only show the model structure for IgM ASCs
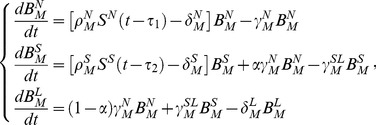
(1)where 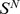 and 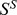 represent the temporal patterns of the synergistic effects on B cell activation in lymph node and spleen, respectively, and their parametric forms are derived from the antigen-presenting cell data [17]–[19] (Supplementary Material S1). Also, we introduce the stimulation strength coefficient (denoted by 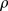) to allow the estimation of the actual magnitude of the stimulation strength [13]. Furthermore, 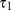 and 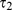 denote the time delay between infection and ASC growth in MLN and spleen, respectively.

**Table 1:** Parameter and variable definitions in Models 1–2 (subscripts for antibody isotypes are not shown).

Variable/Parameter	Definition	Units	Assay	Description
*B^N^*	antigen-specific ASC in MLN	cells per MLN	ELISpot	in all models
*B^S^*	antigen-specific ASC in spleen	cells per spleen	ELISpot	in all models
*B^L^*	antigen-specific ASC in lung	cells per lung	ELISpot	in all models
*S^N^*	B cell activation stimulus (e.g. antigen presenting cells)	cells per MLN	parametric form adapted from literatures [17]–[19]	in Model 1 only
*S^S^*	B cell activation stimulus (e.g. antigen presenting cells)	cells per spleen	parametric form adapted from literatures [17]–[19]	in Model 1 only
*ρ^N^*	stimulation strength coefficent in MLN	day^−1^ per cell per MLN	estimated	in all models
*ρ^S^*	stimulation strength coefficent in spleen	day^−1^ per cell per spleen	estimated	in all models
*δ^N^*	disappearance rate of plasma cells in MLN	day^−1^	estimated	in Model 1 only
*δ^S^*	disappearance rate of plasma cells in spleen	day^−1^	estimated	in Model 1 only
*δ^L^*	disappearance rate of plasma cells in lung	day^−1^	estimated	in all models
*α*	percentage of plasma cells from MLN to spleen	dimensionless	estimated	in all models
*γ^N^*	migration rate of plasma cells out of MLN	day^−1^	estimated	in all models
*γ^SL^*	migration rate of plasma cells from spleen to lung	day^−1^	estimated	in all models
*τ*_1_	lag time for B cell activation in MLN	day	estimated	in all models
*τ*_2_	lag time for B cell activation in spleen	day	estimated	in all models
*S*_(*t*)_	temporal pattern of B cell activation stimulus	cells per organ	nonparametric, estimated	in Model 2 only, time-varing

A concern about the mechanistic model (Model 1) described above is that it may not fully accommodate the complexity of factors that modulate ASC formation and trafficking following influenza infection, and may therefore not sufficiently describe experimental observations. For example, the effects of innate mechanisms such as type I interferon production and complement activation on B cell activation are not explicitly taken into account. In addition, dendritic cell and T cell effects may have different temporal patterns that are not accommodated in Model 1. We therefore considered an alternative semi-mechanistic model (Model 2) in which the terms 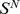 and 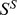 in Model 1 are replaced by the nonparametric time-varying parameter 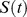 (Supplementary Material S2). Since no explicit assumption is made about the temporal pattern of 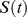, this term can represent the different temporal patterns of innate and adaptive immune mechanisms that drive ASC formation. Model 2 for IgM ASCs is written as
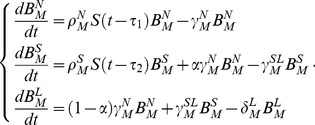
(2)Using the implicit function theorem method [20], one of the standard structural identifiability analysis techniques [21], we verified that the parameters 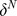 (for MLN) and 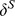 (for spleen) in Model 1 cannot be explicitly included in Model 2. However, 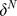 and 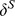 are implicitly included in the terms 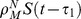 and 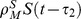, which represent the net rates of increase of ASC populations in MLN and spleen, respectively.

Assumptions and model structures were tested using our experimental data and the statistical methods described below. Mathematical notations and parameter definitions are summarized in Table 1.

### Statistical Methods

We first performed structural identifiability analysis for the ODE models and verified that all parameters were theoretically identifiable [21], [22]. Outliers in the time-course data set of virus-specific ASC numbers for model fitting were automatically identified and removed using the median absolute deviation (MAD) method [23]. Absolute ASC counts represented the difference between counts from virus-coated wells and control wells. Variabilitity resulted in negative ASC counts at some time points and these were assigned a value of zero for analysis since cell counts must be non-negative. Models were fitted to log_10_-transformed data using the robust nonlinear least squares (RNLS) method [24], which is less senstive to data noise (e.g., outliers) than the ordinary NLS. More specifically, instead of the normal distribution assumption, we assumed that the log-transformed data follow a Lorentzian (or Cauchy) distribution and its probability density function is given as
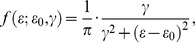
where 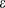 denotes the measurement error, 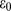 is the location parameter and 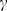 is the scale parameter. In our implementation, the location parameter was fixed as zero and the scale parameter was chosen as the data standard deviation. The Lorentzian distribution has heavier tails so more frequent occurrence of outliers is allowed; therefore, the mean estimation is more robust against outliers than that obtained under the normal distribution assumption. Parameter estimates were obtained using the hybrid optimization algorithm DESQP, which has a superior performance over many alternative methods [25] and has been used in sevearl previous studeis [6], [13], [26], [27]. The 95% confidence intervals for all parameter estimates were calculated using the bootstrap method [28] since the asymptotic confidence intervals calculated using Fisher information matrix (FIM) turned out to be numerically sensitive and unstable. Alternative models were evaluated based on the AICc score, which has a sample size correction and is thus recommended over AIC [29]

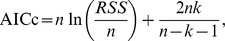
where *RSS* denotes the residual sum of squares, 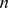 is the number of observations, and 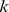 is the number of unknown parameters. A smaller AICc score indicates a better model. Finally, all the computing methods have been implemented and performed in C++ based on the MathStat Library (https://cbim.urmc.rochester.edu/software/mathstat/).

## Results

### Model fitting and evaluation

Experimental data for model fitting was obtained by high frequency sampling during the primary response to intranasal influenza infection. Cell suspensions prepared from MLN, spleen, and lung on days 0 to 14 after infection were analyzed by ELISpot assay to determine virus-specific ASC numbers. ASC counts for samples collected prior to day 4 were generally close to zero in virus-coated and control wells. Because of occasional inconsistencies (deemed as outliers) at early time-points, only data from days 4 to 14 were used for model fitting.

**Figure 2:**
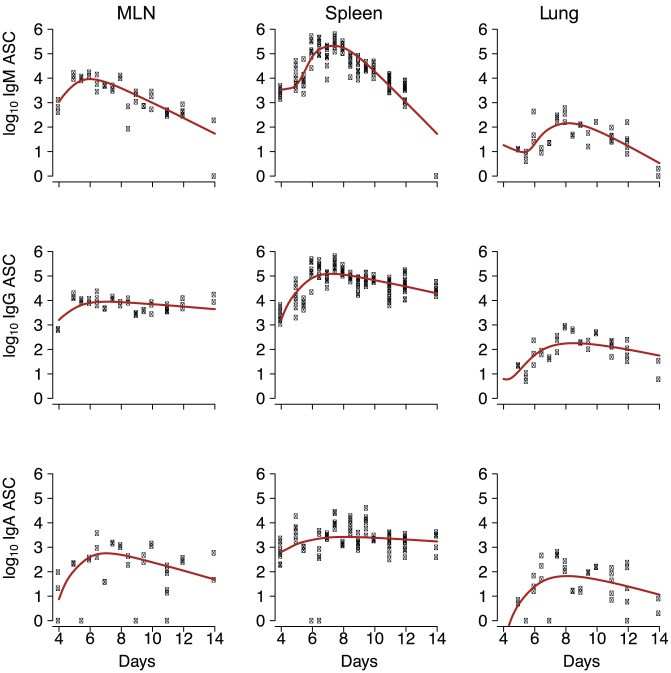
Model fitting results using parameter estimates shown in **Table 2** (derived from Model 1), together with experimental data (individual symbols) plotted against days after infection. Outliers have been removed.

Models 1 and 2 (called the full models, see Table 1 and Fig. 1), together with sub-models derived by setting some parameter values in the full model to zero, were fitted to the experimental data and evaluated based on AICc score [29]. Model 1 outperformed Model 2 for each of the IgM, IgG, and IgA ASC populations, indicating that the increase in ASC populations can be well estimated without introducing the non-parametric form of the synergistic stimulation effects as in Model 2. Point estimates and 95% confidence intervals from Model 1 are presented in Table 2. The 95% confidence intervals were estimated for all model parameters. Curves predicted by Model 1 fitted well with the experimental data (Fig. 2), evidenced by the fact that the *p*-values from the 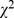 test against the saturated model [30] are greater than 0.3.

**Table 2:** Point estimates based on the best sub-models (derived from Model 1) selected using AICc and the 95% confidence intervals (insignificant parameter estimates selected by AICc are labelled as dropped).

Parameters (unit)	IgM-secreting B cells	IgG-secreting B cells	IgA-secreting B cells
initial *B^N^* cells per MLN	1.1E+3 (2.0E+1, 9.4E+4)	1.6E+3 (7.7E+1, 4.8E+4)	7.6E+0 (1.6E−1, 9.8E+1)
initial *B^S^* cells per spleen	3.4E+3 (3.0E+1, 9.7E+4)	1.6E+3 (5.0E+1, 4.8E+4)	6.5E+2 (2.5E+0, 9.8E+2)
initial *B^L^* cells per lung	1.8E+1 (1.7E+0, 9.4E+3)	6.2E+0 (2.9E−1, 9.8E+2)	0 dropped
*ρ^N^* day^−1^ per cell per MLN	1.3E−4 (4.3E−6, 9.4E−1)	6.6E−5 (2.2E−5, 9.5E−1)	1.0E−4 (NA, 9.6E−1)
*ρ^S^* day^−1^ per cell per spleen	9.6E−5 (2.1E−5, 9.7E−1)	1.1E−4 (3.3E−5, 9.7E−1)	1.9E−5 (NA, 9.5E−1)
*δ^N^* day^−1^	7.3E−1 (1.2E−3, 9.6E+0)	1.2E−1 (3.9E−3, 2.9E+0)	0 dropped
*δ^S^* day^−1^	1.5E+0 (3.4E−3, 9.6E+0)	3.2E−1 (2.2E−3, 2.9E+0)	1.1E−1 (3.0E−2, 4.9E+0)
*δ^L^* day^−1^	8.3E−1 (5.1E−3, 9.8E+0)	7.8E−1 (8.4E−3, 4.8E+0)	9.5E−1 (5.4E−2, 9.4E+0)
*α* (%)	0 dropped	0 dropped	6.8E−1 (2.7E−3, 9.7E−1)
*γ^N^* day^−1^	0 dropped	0 dropped	4.2E−1 (2.4E−2, 9.8E+0)
*γ^SL^* day^−1^	7.6E−4 (5.6E−4, 9.7E−1)	1.4E−3 (4.7E−4, 9.7E−1)	0 dropped
*τ*_1_ day	0 dropped	0 dropped	7.4E−1 (2.6E−2, 5.8E+0)
*τ*_2_ day	2.5E+0 (1.1E−3, 5.7E+0)	6.2E−1 (3.8E−3, 5.8E+0)	1.4E+0 (2.6E−2, 5.8E+0)
*S*_(*t*)_ cells per organ	-	-	-
**RSS**	6.0E+1	3.2E+1	2.0E+2
**AICc**	−8.9E+2	−1.2E+3	−3.4E+2

The lower bound for some highly-skewed parameters are not available and labeled as NA.

### ASC population dynamics in MLN and spleen

The ASC time delay estimates the time from initiation of infection to the initial increase in size of virus-specific ASC populations (Table 3), essentially when the first derivative becomes non-zero. The initial rise in ASC population sizes can be due to either proliferation or differentiation. ASC populations began to increase approximately 1–2 days earlier in MLN than in spleen, consistent with a more rapid delivery of antigen to the MLN via direct lymphatic drainage from the lung.

**Table 3:** Summary of doubling time, half life and half migration time (∞denotes a large value greater than 10^4^, and insignificant parameter estimates selected by AICc are labelled as dropped).

Parameters (unit)	IgM-secreting B cells	IgG-secreting B cells	IgA-secreting B cells
doubling time in MLN (days) 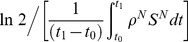	1.6 (2.2E−4, 4.7E+1)	3.1 (2.1E−4, 9.2E+0)	1.1 (6.8E−5, NA)
doubling time in spleen (days) 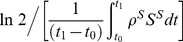	0.64 (6.3E−5, 6.4E+1)	1.2 (7.4E−5, 3.2E+0)	4.1 (6.3E−5, NA)
half-life in MLN (days) 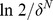	0.94 (1.4E−1, 5.8E+2)	5.7 (2.4E−1, 1.8E+2)	∞
half-life in spleen (days) 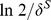	0.46 (7.2E−2, 2.1E+2)	2.2 (2.4E−1, 3.2+2)	6.5 (1.4E−1, 2.3E+1)
half-life in lung (days) 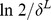	0.84 (7.1E−2, 1.4E+2)	0.89 (1.4E−1, 8.3E+1)	0.73 (7.4E−2, 1.3E+1)
percentage of cells from MLN to spleen (%) *α*	0 dropped	0 dropped	0.68 (2.7E−3, 9.7E−1)
half egress time from MLN (days) 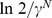	∞	∞	1.7 (7.1E−2, 2.9E+1)
half migration time from spleen to lung (days) 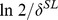	910 (7.1E−1, 1.2E+3)	502 (7.2E−1, 1.5E+3)	∞
stimulation delay in MLN (days) *τ*_1_	0 dropped	0 dropped	0.74 (2.6E−2, 5.8E+0)
stimulation delay in spleen (days) *τ*_2_	2.5 (1.1E−3, 5.7E+0)	0.62 (3.8E−3, 5.8E+0)	1.4 (2.6E−2, 5.8E+0)

The uppper bound for some highly-skewed parameters are not available and labeled as NA.

Parameter values shown in Table 2 were used to calculate measures of ASC population dynamics (Table 3). The results are expressed as *population* doubling times, which are distinct from mitotic duration times of individual cells. Population doubling times were generally shorter for IgM ASCs, consistent with the differentiation and appearance of most of these cells during the early ASC response. Interestingly, the doubling time for the isotype-switched IgA ASC population was substantially shorter in MLN than in spleen, but this was not the case for IgG ASCs. This observation fits with evidence for rapid, T cell-dependent IgA ASC formation in the MLN after influenza infection [2]. As calculated (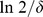), the half-life more precisely represents the half-disappearance time and encompasses ASC population loss from death or functional inactivation and from migration to sites not sampled in this study such as the bone marrow. The half-disappearance time of IgG and IgA ASC populations were shorter in spleen than in MLN, perhaps reflecting rapid ASC exit from the spleen. The relatively short half-disappearance time times of IgG and IgA ASC populations in the lung suggest that many of the early ASC immigrants are short-lived, even though long-lasting resident ASC populations are established in the lung [3], [4], [31].

### ASC migration from MLN and spleen

**Figure 3:**
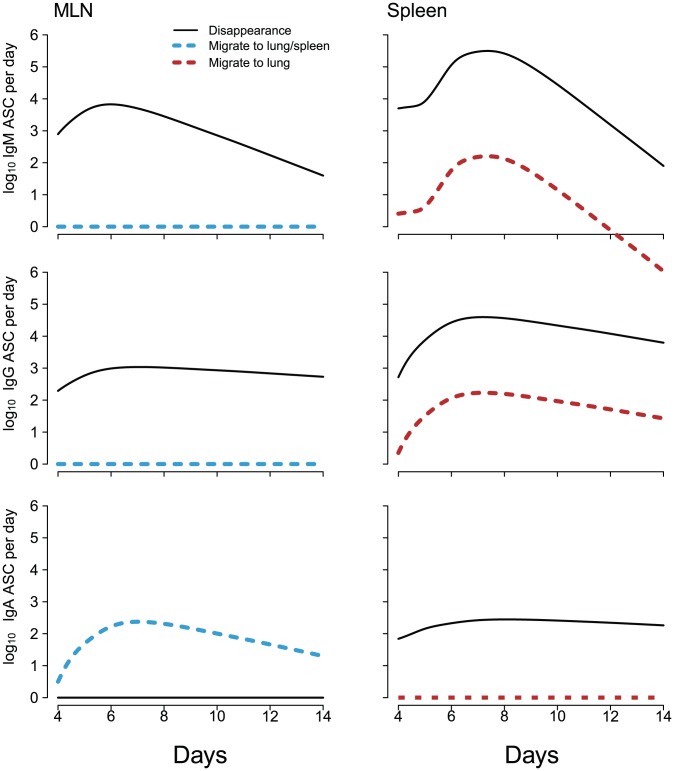
ASC disappearance compared with ASC migration from MLN and spleen. The term migration covers ASC trafficking from MLN to lung or spleen, or ASC trafficking from spleen to lung. Migration pathways reflect assumptions shown in Figure 1. The term disappearance covers ASC death or ASC migration to sites other than the lung or spleen.

To assess the characteristics of ASC loss from MLN and spleen, estimated migration rates and disappearance rates of ASC populations were plotted against sampling time (Fig. 3). Our assumptions related to migration pathways from MLN and spleen were described in the previous section (see Fig. 1). Disappearance essentially accounted for all IgM and IgG ASC loss from the MLN, with negligible loss occurring through migration to spleen or lung. In contrast, IgA ASC loss from the MLN primarily resulted from migration to spleen or lung and not from disappearance. Disappearance accounted for most of the IgM, IgG, and IgA ASC loss from the spleen, although there was some IgM and IgG ASC migration from spleen to lung. These observations fit with the estimated half-migration times for ASC populations exiting the MLN and for ASC populations migrating from spleen to lung (Table 3). The half-migration time for the IgA ASC population in the MLN was only 1.7 days, but was substantially longer for IgM and IgG ASC populations. Half-migration times for trafficking to the lung were relatively long for all ASC populations in the spleen. This was particularly the case for the IgA ASC population, consistent with minimal IgA ASC migration from spleen to lung.

The difference between MLN and spleen in their contribution to ASC populations in the lung is also shown in Fig. 3. Interestingly, the MLN contributed most of the lung IgA ASCs, even though the spleen contained substantial numbers of IgA ASCs. In contrast, IgM and IgG ASCs that localized in the lung were mainly from the spleen, even though relatively strong IgM and IgG responses were generated earlier in the MLN. Overall, these observations point towards fundamental differences between MLN and spleen in features of the B cell response to influenza infection.

**Figure 4:**
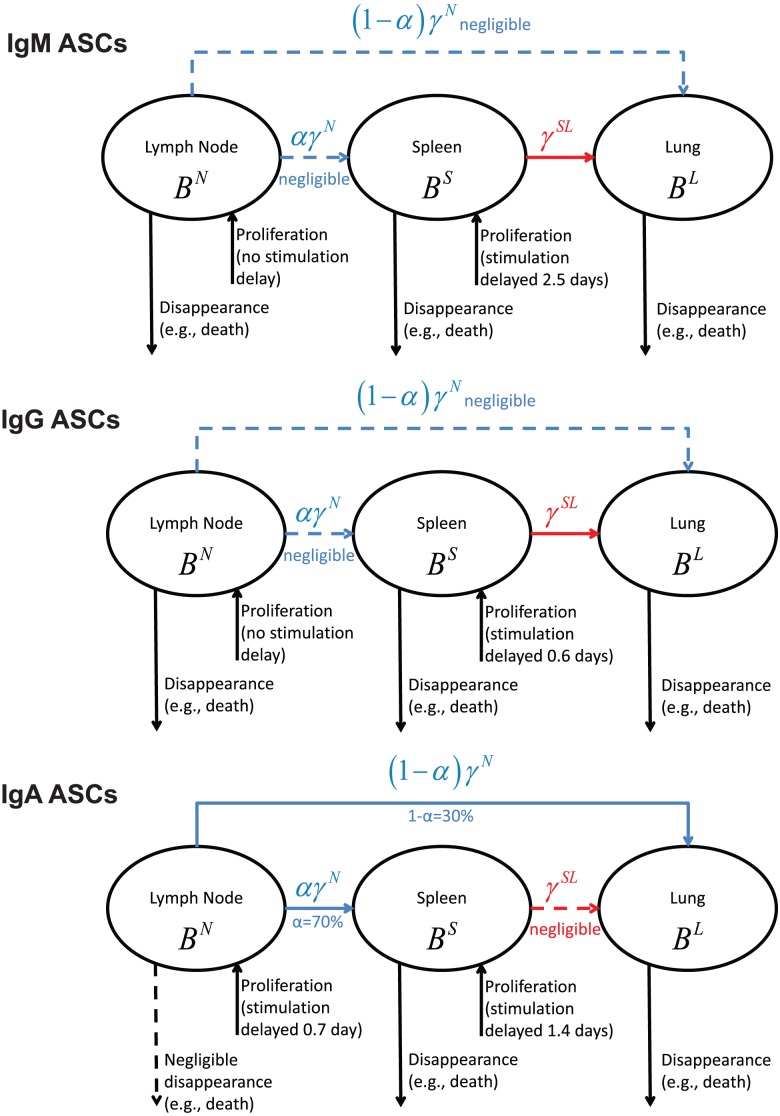
Visualization of the modeling results for the three types of ASCs, where the solid lines represent statistically significant parameter estimates and the dashed lines are for negligible parameter values.

Finally, for convenience, the modeling results are summarized and visualized in Fig. 4 for three types of ASCs considered in this study. The solid lines in Fig. 4 represent statistically significant parameter estimates, and the dashed lines are for negligible model terms.

### In silico validation

A key question was whether limitations in the experimental data would significantly impact model predictions. In our analysis, the lymph node compartment was represented by ASC response data from the MLN, the major lymph node draining the lower respiratory tract. However, influenza X31 infection also elicits substantial ASC formation in lymph nodes draining the upper respiratory tract, predominantly in the group of cervical lymph nodes (CLN). The CLN are approximately 10-fold more cellular than the MLN throughout the response to X31 infection and, based on data from other studies, are likely to contribute up to 10-fold more virus-specific ASCs than does the MLN [4], [32], [33]. A limited analysis of virus-specific ASCs on days 5, 7, and 9 after influenza X31 infection demonstrated that the ratios of IgM, IgG, and IgA ASCs were similar in the CLN and MLN (Fig. S1 in Supporting Information). Another potential issue in our data was underestimation of the number of virus-specific ASCs that migrate to the lung. The results of other studies suggest that the use of an enzymatic digestion protocol might have increased our recovery of ASCs from lung tissue by up to 10-fold [1], [3].

We therefore assessed the robustness of our best models (Table 2) to a combination of (i) a 10-fold increase in the number of ASCs generated in the lymph node compartment, and (ii) a 10-fold increase in the number of ASCs isolated from the lung. These increases were applied to the numbers of IgM, IgG, and IgA ASCs. The results of model fitting (Fig. S2 in Supporting Information) showed that all measures of ASC population dynamics shown in Table 3 were only marginally changed when calculated using the adjusted ASC counts. The plots shown in Fig. 3 also had the same patterns when the adjusted data set was used (Fig. S3 in Supporting Information). Recently, Anderson and colleagues [34] demonstrated that a high proportion of lymphocytes recovered from the perfused lung were located in the vasculature rather than in the lung tissue itself. Although a similar situation for ASCs has not been established, we also asked whether an overestimation of the number of ASCs that entered lung tissue would impact model predictions. We tested the robustness of our models using a 2-fold decrease in lung IgM, IgG, and IgA ASCs and a 10-fold increase in ASCs from the lymph node compartment. Model predictions in Fig. 3 were unchanged using the adjusted data sets. In summary, the in silico testing demonstrated that model predictions were unaffected by increased ASC numbers generated in the lymph node compartment in combination with an increase or decrease in lung ASC numbers.

We also conducted a sensitivity analysis by calculating the changes in model outputs (that is, the areas under the curves for 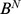, 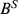 and 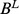) corresponding to 1%, 5% or 10% changes in parameter values [35]. The results indicate that model outputs are not sensitive to perturbations in any parameter except for 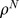 (a 50% change in model outputs was observed for a 10% change in 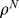). However, note that 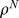 primarily affects the magnitudes of ASC numbers in the lymph node and lung compartments and not ASC trafficking routes.

## Discussion

Numerous studies have provided a general picture of the kinetics of virus-specific ASC formation and migration following primary influenza infection [1]–[5], but the quantitative dynamics of ASC populations have received little attention. For example, the relative population doubling times for IgM, IgG, and IgA ASCs have not been estimated, nor have their trafficking patterns during infection been studied in detail. In particular, the “burst size” or total number of virus-specific ASCs generated during the response in various lymphoid and non-lymphoid tissues has rarely been addressed. Part of the issue has been the difficulty in direct experimental measurement of population doubling times. We have addressed these issues by constructing a compartmental model that, for the first time, estimates ASC trafficking patterns, population doubling rates, and rates of disappearance from cell death, quiescence, or migration to other compartments.

Before discussing detailed model findings, it is important to recognize that estimates of population doubling times provide different and more informative information than estimates of activated ASC division times. There is much heterogeneity in B cell activation, differentiation and death times in immune responses. Thus, one population doubling time could model many different combinations of cells with different division times, without a way to discern the actual mixture experimentally. In order to estimate the total Ab secretion of ASCs contributing to influenza-specific serum Ab, one must have detailed information regarding the population heterogeneity and estimate several more parameters, integrating Ab secretion rates over the time of the immune response. Experimental validation of these additional parameters is quite difficult *in vivo*. In contrast, estimates of influenza-specific ASC population doubling time can be used directly to estimate changes in Ab concentration over time with a single parameter, and fewer identifiability issues. This actually leads to greater model precision, and is the primary reason we chose this parameterization of our model.

Our modeling estimates of population doubling times for IgM ASCs in MLN and spleen fit with the well-established concept of rapid IgM ASC formation preceding the IgG response. Interestingly, the population doubling time for IgA ASCs was comparable to that for IgM ASCs in the MLN but not in the spleen. This finding is consistent with previous studies that reported a rapid T cell-dependent IgA ASC response preceding IgG production in the MLN of influenza-infected mice [2], [5]. Although both the IgA and IgG responses in this model of influenza infection are T cell-dependent, there are apparently key differences in the mechanisms of delivery of T cell help. In contrast to the situation for IgG production, T cell help for the early IgA response can be delivered in a non-antigen-specific manner to B cells deficient in major histocompatibility complex class II [2]. This may relate to the rapidity of the IgA response, but the mechanism is not understood. Early IgA ASCs in the MLN are almost exclusively specific for viral surface glycoproteins, with IgA ASCs specific for the internal nucleocapsid protein only appearing in the second week after infection [5]. Taken together, these observations have led to the proposal that the early IgA response is initiated when specific B cell clones recognize viral surface glycoproteins on infected DCs and is driven by soluble differentiation and class switching factors without the need for cognate T cell help [2], [5].

One potential drawback to our model is our use of composite parameters for cell migration and disappearance. We use the term migration to include ASC trafficking from the MLN to spleen or lung, and from the spleen to lung; the term disappearance encompasses ASC loss from cell death, as well as migration to sites not sampled in our analysis. We used the composite parameter disappearance because both statistical and experimental methods were not able to separately estimate *in vivo* cell death and migration to other sites in multiple compartments. Accurately measuring *in vivo* cell death is difficult given the rapid clearance of apoptotic and necrotic cells [36]. Accounting for migration of ASCs to all unmeasured compartments is a difficult experimental challenge, requiring transgenic mouse systems and whole mouse imaging [37]. Definitive experiments would involve murine infection followed by simultaneous sampling of all lymph nodes within BcR transgenic mice to account for migration to non-draining lymph nodes, nasal mucosa and other mucosa-associated lymphoid tissues, as well as skin and liver. Novel radioactive labeling and imaging methods could be used as well. The numbers of mice and technical challenges for simultaneous daily collection of such data makes this a point of experimental investigation for future projects. However, the current manuscript demonstrates that the use of a composite parameter allows us to narrow down the mechanisms of cell disappearance while preserving model identifiability.

From about 7 days after influenza infection, virus-specific ASCs can be isolated from the tissues of the upper and lower respiratory tract. IgA ASCs predominate in the upper respiratory tract and are also numerous in the lung, together with IgG and (less consistently) IgM ASCs [3], [4]. It is generally accepted that ASCs generated by influenza infection migrate to the respiratory tract from sites of formation in organized lymphoid tissues. Our modeling estimates extend this concept and indicate differences in ASC trafficking patterns that depend on site of formation and the expressed Ab class. For ASC populations in MLN and spleen, we estimated loss from migration or from disappearance. Notably, our modeling suggests that essentially all IgA ASC loss from the MLN results from migration to spleen or lung, with negligible IgA ASC migration from spleen to lung. This result points to a critical role for regional lymph nodes such as the MLN in the rapid generation of IgA ASCs that migrate to the respiratory tract in the course of influenza infection. These ASCs presumably take up residence in the lamina propria of the respiratory tract and secrete IgA that is transcytosed across epithelial cells and released at the mucosal surface. The kinetics of IgA ASC localization in the respiratory tract suggests that they contribute to the clearance of infectious virus. IgA with virus-neutralizing activity has been identified in nasal washings from mice on day 7 after influenza infection, a time when viral titers in the respiratory tract are declining [2], [4].

In contrast to IgA ASCs, essentially all IgM and IgG ASC loss from the MLN occurred through disappearance and not from migration to spleen or lung. It seems unlikely that significant IgM and IgG ASC loss from the MLN during the period of our analysis resulted from migration to sites that were not sampled (encompassed by our use of the term disappearance). In addition to the respiratory tract, the major target site for ASC migration during the B cell response to influenza infection is the bone marrow. However, virus-specific ASCs are generally not detected in the bone marrow until 10 days or more after infection of the respiratory tract [4], [14], [38]. This points to cell death as the major reason for IgM and IgG ASC loss from the MLN. Presumably, these cells are products of the T cell-dependent extrafollicular pathway, which generates short-lived ASCs from activated B cells prior to the development of germinal centers. This is supported by evidence that virus-specific IgM, IgG, and IgA ASCs are present in the MLN before germinal center formation is first evident approximately 7–8 days after influenza infection [5], [39], [40]. Antiviral Abs produced via the extrafollicular pathway provide an early barrier to the spread of infection and also facilitate further virus-specific B cell activation [41].

Conventional dogma has cell death coinciding with contraction of the immune response. Our model suggests that ASC death begins early and continues throughout the B cell response. Our estimates indicate a more rapid death rate for IgM ASCs than for IgG ASCs, perhaps reflecting a large component of IgM ASC formation in the short-lived extrafollicular response or a survival advantage associated with IgG class switching signals. Apparently, the early IgA ASCs in the MLN are not susceptible to cell death like the IgM and IgG ASCs and, instead, leave the MLN and migrate to other sites. This fits with evidence discussed above that points to a mechanism of early IgA ASC generation that is distinct from the standard extrafollicular differentiation pathway [2], [5]. Signals delivered to the developing IgA ASCs may promote survival and the expression of mucosal homing molecules [42].

Our modeling results suggest that the majority of IgM and IgG ASCs that migrate to the lung come from the spleen and not the MLN. This contrasts with the situation for IgA ASCs in the lung, most of which come from the MLN and not the spleen. An important factor may be the relative magnitude of the germinal center response in the spleen compared with the MLN. At the time of initial germinal center formation in the MLN and spleen on approximately day 8 after infection, the frequency of germinal center B cells (percentage of total cells) is similar in both locations. However, because of the cellularity of the spleen, this represents approximately 50-fold more germinal center B cells in the spleen [40]. It follows that the spleen is likely to generate substantial numbers of long-lived ASCs via the germinal center pathway at a stage of the response when the majority of IgM and IgG ASCs formed in the MLN are short-lived. ASCs generated in germinal centers typically express CXCR4 and traffic from sites of formation to the bone marrow where long-lasting populations are established. In addition, CXCR4-expressing ASCs may traffic to sites of inflammation, such as the influenza-infected lung, in response to local production of inflammatory chemokines. In particular, this may apply to a subset of IgM and IgG ASCs that express CXCR3, consistent with our modeling results [43], [44]. The rates of ASC disappearance from the spleen measured in our analysis likely encompass not only cell death, as in the MLN, but also ASC movement into the circulation to seed the bone marrow. At times in our analysis, substantial numbers of IgA ASCs were present in the spleen, but our modeling indicated minimal migration of these cells from spleen to lung. The reason for this is unclear. Although some IgA ASCs in the spleen may have been generated by germinal center reactions, others with limited further migration potential may have trafficked to the spleen after formation in the MLN.

Our estimates of the half-life (more precisely the half-disappearance time) of ASC populations in the lung essentially only reflect cell death, since ASC migration from the lung is probably minimal. ASC migration to the lung in response to influenza infection establishes long-lasting populations [3], [4], [31]. It is therefore interesting that our half-disappearance time estimates for IgM, IgG, and IgA ASC populations in the lung were relatively short, indicating that these cells are not inherently long-lived. The longevity of ASCs in the lung, as in the bone marrow, may depend on a stable and supportive tissue environment that provides appropriate anchoring sites and survival factors [45]. Such an environment may not present in the lung during the periods of inflammation and tissue repair. Our modeling is consistent with a continuous influx of ASCs into the lung to maintain ASC population sizes, at least for a period of time after infection.

In summary, we have combined high frequency experimental data and mathematical modeling to provide estimates of ASC population dynamics that are difficult to measure experimentally. The results of our analysis of virus-specific ASC populations generated by primary influenza infection of the respiratory tract support existing concepts and, in addition, provide novel insights and identify directions for future experimentation. Most notably, our findings (i) emphasize the importance of the MLN in the early generation of IgA ASCs that migrate to the lung, (ii) point to key differences between mechanisms of IgA ASC formation and those of IgM and IgG ASC formation in the MLN that influence the longevity and migratory potential of early ASCs, and (iii) identify differences between MLN and spleen in their contribution to particular ASC populations in the lung. Further studies are required to provide a mechanistic understanding of the increasingly complex picture of ASC dynamics and trafficking that is emerging.

## Supporting Information

Figure S1 **Virus-specific ASC formation in the MLN and CLN following influenza infection.** (A, B) Virus-specific ASC frequencies. (C, D) Proportions of virus-specific ASCs producing the IgM, IgG, and IgA Ab classes. B6 mice were infected intranasally with 10^5^ EID_50_ of influenza X31. Virus-specific ASCs were enumerated by ELISpot assay at intervals after infection. The mean + SD is shown for 3–5 individual mice per group.
(DOCX)

Figure S2 **Fitted curves corresponding to a 10-fold increase in the ASC numbers in lymph node and lung.**
(DOCX)

Figure S3 **ASC disappearance compared with ASC migration from MLN and spleen after a 10-fold increase in the ASC numbers in lymph node and lung.**
(DOCX)

Text S1 **Derivation of the parametric form of a time-varying variable from data.**
(DOCX)

Text S2 **Definition and use of a nonparametric time-varying parameter.**
(DOCX)
