# Supplementary material for: Modeling the Dynamics and Migratory Pathways of Virus-Specific Antibody-Secreting Cell Populations in Primary Influenza Infection
Source: PLoS One. 2014 Aug 29;9(8):e104781. doi: 10.1371/journal.pone.0104781 (PMC4149352; doi:10.1371/journal.pone.0104781)
Supplement: Figure S1 — Virus-specific ASC formation in the MLN and CLN following influenza infection. (A, B) Virus-specific ASC frequencies. (C, D) Proportions of virus-specific ASCs producing the IgM, IgG, and IgA Ab classes. B6 mice were infected intranasally with 105 EID50 of influenza X31. Virus-specific ASCs were enumerated by ELISpot assay at intervals after infection. The mean + SD is shown for 3–5 individual mice per group. (DOCX) [file pone.0104781.s001.docx]

**Figure S1.** Virus-specific ASC formation in the MLN and CLN following influenza infection. (A, B) Virus-specific ASC frequencies. (C, D) Proportions of virus-specific ASCs producing the IgM, IgG, and IgA Ab classes. B6 mice were infected intranasally with 10^5^ EID_50_ of influenza X31. Virus-specific ASCs were enumerated by ELISpot assay at intervals after infection. The mean + SD is shown for 3-5 individual mice per group.
