# Supplementary material for: Modeling the Dynamics and Migratory Pathways of Virus-Specific Antibody-Secreting Cell Populations in Primary Influenza Infection
Source: PLoS One. 2014 Aug 29;9(8):e104781. doi: 10.1371/journal.pone.0104781 (PMC4149352; doi:10.1371/journal.pone.0104781)
Supplement: Figure S3 — ASC disappearance compared with ASC migration from MLN and spleen after a 10-fold increase in the ASC numbers in lymph node and lung. (DOCX) [file pone.0104781.s003.docx]

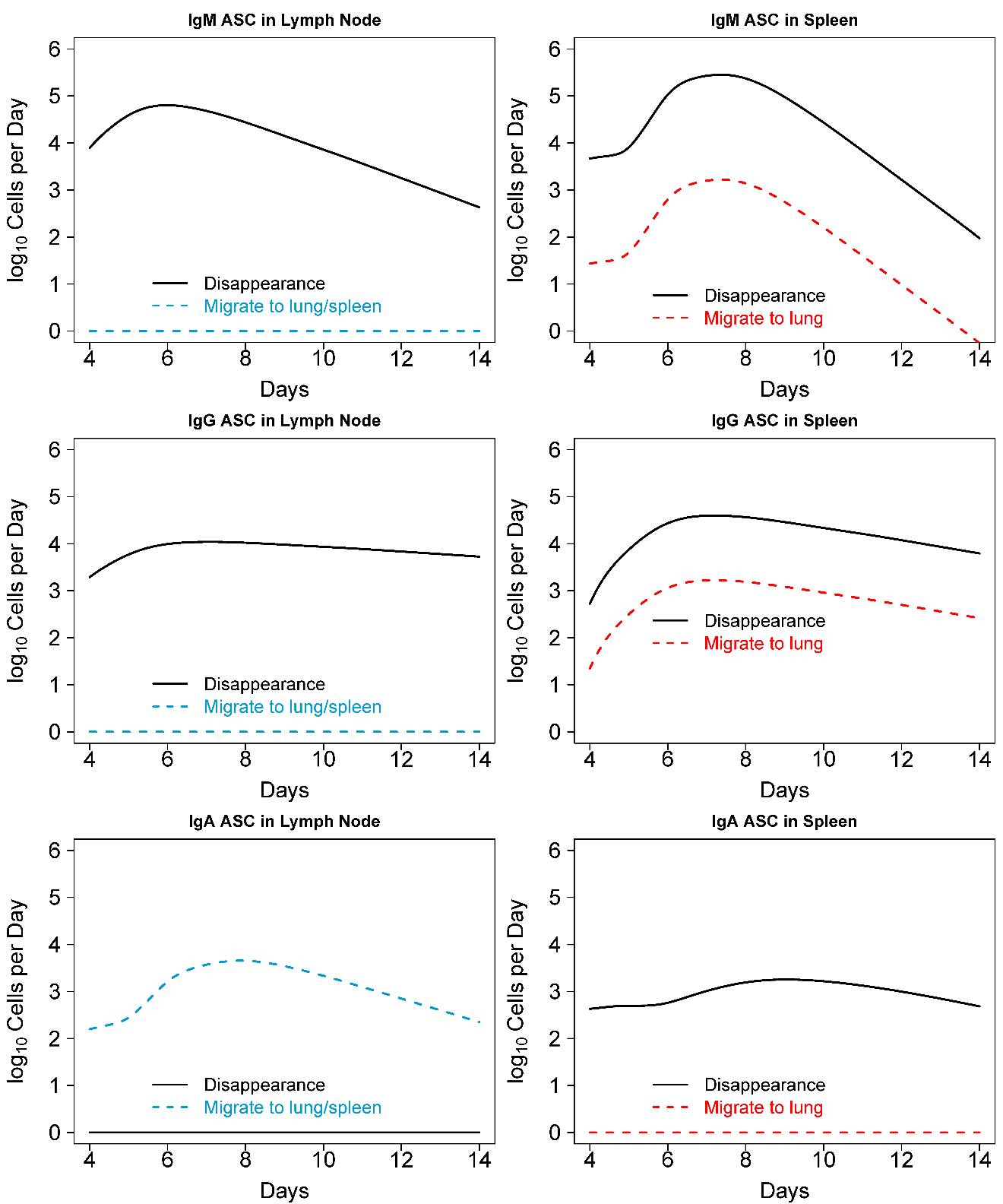
­­

**Figure S3**. ASC disappearance compared with ASC migration from MLN and spleen after a 10-fold increase in the ASC numbers in lymph node and lung.
