# Supplementary material for: Modeling the Dynamics and Migratory Pathways of Virus-Specific Antibody-Secreting Cell Populations in Primary Influenza Infection
Source: PLoS One. 2014 Aug 29;9(8):e104781. doi: 10.1371/journal.pone.0104781 (PMC4149352; doi:10.1371/journal.pone.0104781)
Supplement: Text S1 — Derivation of the parametric form of a time-varying variable from data. (DOCX) [file pone.0104781.s004.docx]

**Text S1. Derivation of the parametric form of a time-varying variable from data.**

The time-varying variable *S*(*t*) in Model (1) is a smooth function of time and acts as an input to the model. Note that *S*(*t*) cannot be directly solved from the differential equation model, so we assume that it can be determined from, e.g., experiment data. However, real data always have measurement error; to determine a smooth curve from data, we thus seek the use of cubic smoothing spline.

That is, $S\left(t\right)$ can be approximated by the linear combination of the basis-spline functions,

$S\left(t\right)=\sum_{j=1}^{q} a_{j}b_{j,k}\left(t\right)$, (1)

where $a_{1},a_{2},...,a_{q}$are the unknown coefficients to be determined, $q$ the degrees of freedom, and $b_{j,k}\left(t\right)$ the basis function of a pre-specified order $k$ (usually *k*=4, called cubic spline bases). In addition, let $y_{i}$ denote the observations for $S\left(t\right)$collected at time points $t_{i},i=1,2,...,n$. The smooth curve $\hat{S}\left(t\right)$ is then estimated to achieve the optimal balance between smoothness and fitness by minimizing the following objective function:

$R\left(\lambda\right)=\sum_{i=1}^{n} \left[y_{i}-S\left(t_{i}\right)\right]^{2}+\lambda\int\left[S''\left(t\right)\right]^{2}dt$, (2)

where $\lambda\geq0$ is the penalty coefficient. Substitute Eq. (1) into Eq. (2) to obtain

$R\left(a,\lambda\right)=\left(Y-Ba\right)^{T}\cdot\left(Y-Ba\right)+\lambda a^{T}\cdot M\cdot a$, (3)

where $M=\int b''b''^{T}dt$ and

$a=\left[\begin{matrix} a_{1} \\ a_{2} \\ \vdots\\ a_{q} \end{matrix}\right]_{q\times1}$, $b=\left[\begin{matrix} b_{1,k}\left(t\right) \\ b_{2,k}\left(t\right) \\ \vdots\\ b_{q,k}\left(t\right) \end{matrix}\right]_{q\times1}$, $Y=\left[\begin{matrix} y_{1} \\ y_{2} \\ \vdots\\ y_{n} \end{matrix}\right]_{n\times1}$, $B=\left(\begin{matrix} b_{1,k}\left(t_{1}\right) & b_{2,k}\left(t_{1}\right) & \cdots& b_{q,k}\left(t_{1}\right) \\ b_{1,k}\left(t_{2}\right) & b_{2,k}\left(t_{2}\right) & \cdots& b_{q,k}\left(t_{2}\right) \\ \vdots& \vdots& \vdots& \vdots\\ b_{1,k}\left(t_{n}\right) & b_{2,k}\left(t_{n}\right) & \cdots& b_{q,k}\left(t_{n}\right) \end{matrix}\right)_{n\times q}$ .

According to Eq. (3), the estimate of $a$ can be obtained as follows

$\hat{a}=\left(B^{T}B+\lambda M\right)^{-1}B^{T}Y$, (4)

and the penalty coefficient $\lambda$ can be determined using the generalized cross validation (GCV)

$GCV\left(\lambda\right)=\frac{\frac{1}{n}\left\|(I-H)\cdot Y\right\|^{2}}{\left[\frac{1}{n}tr\left(I-H\right)\right]^{2}}$ , (5)

where $H=B\left(B^{T}B+\lambda M\right)^{-1}B^{T}$is the so called hat matrix.

Smoothing splines have been implemented in a number of mainstream computing environments such as MATLAB® and R. In this study, we used the *smooth.spline* routine in R.
