# Supplementary material for: Modeling the Dynamics and Migratory Pathways of Virus-Specific Antibody-Secreting Cell Populations in Primary Influenza Infection
Source: PLoS One. 2014 Aug 29;9(8):e104781. doi: 10.1371/journal.pone.0104781 (PMC4149352; doi:10.1371/journal.pone.0104781)
Supplement: Text S2 — Definition and use of a nonparametric time-varying parameter. (DOCX) [file pone.0104781.s005.docx]

**Text S2. Definition and use of a nonparametric time-varying parameter.**

The time-varying coefficient *S*(*t*) in Model (2) is also a smooth function of time and acts as an input to this model. However, instead of assuming the curve *S*(*t*) is equivalent to the smoothed curve from the time-course dendritic cell counts (Text S1), *S*(*t*) is now allowed to take any shape such that *S*(*t*) can also accommodate the effects of factors other than dendritic cells on ASCs. For this purpose, we can still use the basis-spline approximation

$S\left(t\right)=\sum_{j=1}^{q} a_{j}b_{j,k}\left(t\right)$,

but now we substitute this approximation into the differential equation model to obtain

$\left\{\begin{matrix} \frac{dB_{M}^{N}}{dt}=\rho_{M}^{N}\cdot\left[\sum_{j=1}^{q} a_{j}b_{j,k}\left(t-\tau_{1}\right)\right]\cdot B_{M}^{N}-\gamma_{M}^{N}B_{M}^{N} \\ \frac{dB_{M}^{S}}{dt}=\rho_{M}^{S}\cdot\left[\sum_{j=1}^{q} a_{j}b_{j,k}\left(t-\tau_{2}\right)\right]\cdot B_{M}^{S}+\alpha\gamma_{M}^{N}B_{M}^{N}-\gamma_{M}^{SL}B_{M}^{S} \\ \frac{dB_{M}^{L}}{dt}=\left(1-\alpha\right)\gamma_{M}^{N}B_{M}^{N}+\gamma_{M}^{SL}B_{M}^{S}-\delta_{M}^{L}B_{M}^{L} \end{matrix}\right.$.

Thus, the linear coefficients $\left(a_{1},a_{2},...,a_{q}\right)$ become part of the unknown ODE model parameters like $\left(\alpha,\rho_{M}^{N},\rho_{M}^{S},\gamma_{M}^{N},\gamma_{M}^{SL},\delta_{M}^{L}\right)$. Altogether, $\left(a_{1},a_{2},...,a_{q},\alpha,\rho_{M}^{N},\rho_{M}^{S},\gamma_{M}^{N},\gamma_{M}^{SL},\delta_{M}^{L}\right)$ are the unknown parameter vector and can be determined from ASCs data using least squares. Note that the procedure described above is very different from what is in Text S1; also, the time-course dendritic cell counts used in Text S1 are not used here.
